# Machine learning analysis of humoral and cellular responses to SARS-CoV-2 infection in young adults

**DOI:** 10.3389/fimmu.2023.1158905

**Published:** 2023-05-29

**Authors:** Ricards Marcinkevics, Pamuditha N. Silva, Anna-Katharina Hankele, Charlyn Dörnte, Sarah Kadelka, Katharina Csik, Svenja Godbersen, Algera Goga, Lynn Hasenöhrl, Pascale Hirschi, Hasan Kabakci, Mary P. LaPierre, Johanna Mayrhofer, Alexandra C. Title, Xuan Shu, Nouell Baiioud, Sandra Bernal, Laura Dassisti, Mara D. Saenz-de-Juano, Meret Schmidhauser, Giulia Silvestrelli, Simon Z. Ulbrich, Thea J. Ulbrich, Tamara Wyss, Daniel J. Stekhoven, Faisal S. Al-Quaddoomi, Shuqing Yu, Mascha Binder, Christoph Schultheiβ, Claudia Zindel, Christoph Kolling, Jörg Goldhahn, Bahram Kasmapour Seighalani, Polina Zjablovskaja, Frank Hardung, Marc Schuster, Anne Richter, Yi-Ju Huang, Gereon Lauer, Herrad Baurmann, Jun Siong Low, Daniela Vaqueirinho, Sandra Jovic, Luca Piccoli, Sandra Ciesek, Julia E. Vogt, Federica Sallusto, Markus Stoffel, Susanne E. Ulbrich

**Affiliations:** ^1^ Department of Computer Science, ETH Zurich, Zurich, Switzerland; ^2^ Institute of Molecular Health Sciences, ETH Zurich, Zurich, Switzerland; ^3^ Animal Physiology, Institute of Agricultural Sciences, ETH Zurich, Zurich, Switzerland; ^4^ Miltenyi Biotec B.V. & Co. KG, Bergisch Gladbach, Germany; ^5^ Institute of Integrative Biology, ETH Zurich, Zurich, Switzerland; ^6^ NEXUS Personalized Health Technologies, Zurich & SIB Swiss Institute of Bioinformatics, ETH Zurich, Lausanne, Switzerland; ^7^ Department of Internal Medicine IV, Oncology/Hematology, Martin-Luther-University Halle-Wittenberg, Halle (Saale), Germany; ^8^ Department of Health Science, Translational Medicine, ETH Zurich, Zurich, Switzerland; ^9^ Institute for Research in Biomedicine, Università della Svizzera Italiana, Bellinzona, Switzerland; ^10^ Humabs BioMed SA, a Subsidiary of Vir Biotechnology, Bellinzona, Switzerland; ^11^ Institute of Medical Virology, Goethe University Frankfurt, Frankfurt am Main, Germany; ^12^ Medical Immunology, Institute of Microbiology, ETH Zurich, Zurich, Switzerland; ^13^ University Hospital Zurich, Zurich, Switzerland

**Keywords:** SARS-CoV-2, antibody titers, T cell response, machine learning, neutralizing antibodies

## Abstract

The severe acute respiratory syndrome coronavirus 2 (SARS-CoV-2) induces B and T cell responses, contributing to virus neutralization. In a cohort of 2,911 young adults, we identified 65 individuals who had an asymptomatic or mildly symptomatic SARS-CoV-2 infection and characterized their humoral and T cell responses to the Spike (S), Nucleocapsid (N) and Membrane (M) proteins. We found that previous infection induced CD4 T cells that vigorously responded to pools of peptides derived from the S and N proteins. By using statistical and machine learning models, we observed that the T cell response highly correlated with a compound titer of antibodies against the Receptor Binding Domain (RBD), S and N. However, while serum antibodies decayed over time, the cellular phenotype of these individuals remained stable over four months. Our computational analysis demonstrates that in young adults, asymptomatic and paucisymptomatic SARS-CoV-2 infections can induce robust and long-lasting CD4 T cell responses that exhibit slower decays than antibody titers. These observations imply that next-generation COVID-19 vaccines should be designed to induce stronger cellular responses to sustain the generation of potent neutralizing antibodies.

## Introduction

Severe acute respiratory syndrome coronavirus 2 (SARS-CoV-2) is the etiological agent of the coronavirus disease 2019 (COVID-19). Both humoral and cellular immune responses against SARS-CoV-2 have major implications for the clinical outcome of COVID-19, the risk of reinfection and the efficacy of vaccination.

Antibodies are produced from plasma cells. The B cell maturation into plasma cells is supported by CD4 T cells *via* cell-cell interactions and cytokine secretion, while CD8 T cells eliminate virus-infected cells through cytolytic activity. B and T cells are therefore essential for eliminating the virus and establishing immunological memory. However, the extent to which T cell responses contribute to SARS-CoV-2 clearance and, more importantly, long-term protection is still under investigation. Early studies with human subjects have reported that COVID-19 patients with X-linked or autosomal-recessive agammaglobulinemia were able to recover from infection without severe disease requiring intensive care (1), however, subsequent studies also reported increased respiratory viral detection and symptom burden among patients with primary antibody deficiency (2). While B cells are critical for preventing infection or reducing inoculum size, T cell responses play a prominent role in clearing the infection (3, 4). Furthermore, immune responses to SARS-CoV-2 that generate coordinated CD4 and CD8 T cell-based immunity have been shown to correlate with favorable outcomes in COVID-19 patients (5–7). COVID-19 patients with B cell immunodeficiencies showed favorable outcomes upon strong CD8 T cell responses (8). These findings underline the importance of CD8 T cell mediated cytotoxicity in viral clearance, potentially contributing to a milder disease course. Together, these observations indicate that T cells provide substantial protective immunity, which limits severe disease in settings where antibody responses are diminished, thereby being beneficial for COVID-19 patients.

In this study, we analyzed *via* machine learning (ML) and classical statistical modeling SARS-CoV-2 specific humoral and T cell immune responses in a cohort of young convalescent adults with asymptomatic or mildly symptomatic SARS-CoV-2 infections. We aimed to assess if particular viral antigens induce antibody- and/or cell-mediated immunodominance. To this end, we determined which T cell subset and activation marker combinations allow predicting the antibody status. Furthermore, we employed ML methods in addition to conventional statistical modeling to uncover potentially nonlinear and complex associations among humoral and T cell immune responses. Our integrated approach to studying B cell, CD4 and CD8 T cell responses to SARS-CoV-2 allowed us to identify associations between the class of immune cells responding to SARS-CoV-2 and the virus components triggering such responses. Finally, we explored associations between T cell responses of COVID-19 patients and self-reported symptoms scores.

## Materials and methods

### CoV-ETH cohort

The ethical approval for the CoV-ETH study (CoV-ETH cohort) was obtained from the Cantonal Ethics Commission Zurich (BASEC-Nr. 2020-00949). Written informed consent was received from all participants. The study has been performed in accordance with the Declaration of Helsinki of 1975.

**Figure 1:**
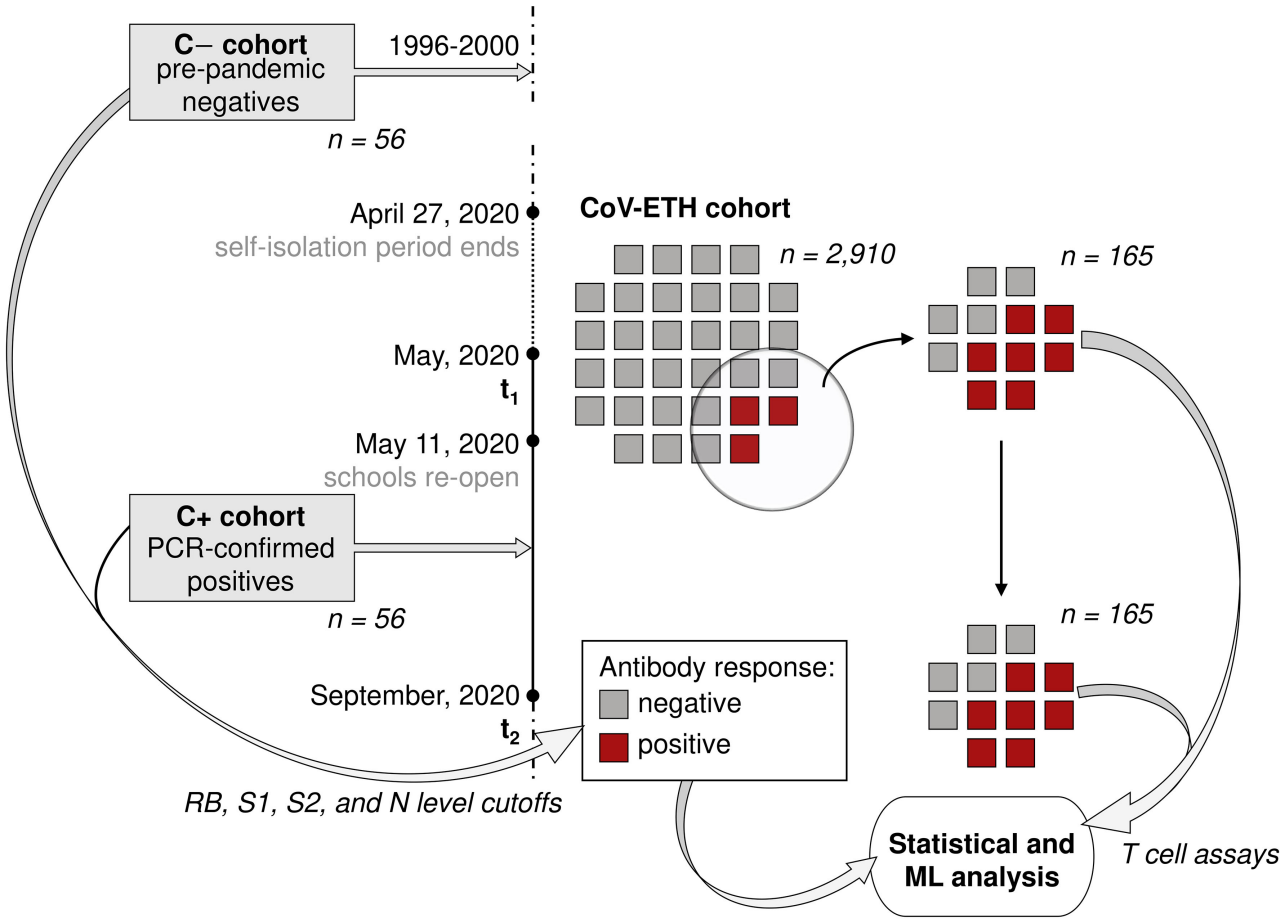
Study overview. The CoV-ETH study was launched in May 2020 and comprised of 2,911 individuals without previous knowledge on SARS-CoV-2 immune state. A serological screening assessed 65 seroconverted individuals at the first screening in May 2020. From these and 69 randomly chosen non-seroconverted negative controls, blood samples from May and September 2020 were analyzed for humoral and T cell response. As positive and negative controls, 56 samples from 36 PCR-confirmed hospitalized SARS-CoV-2 infected individuals and pre-pandemic samples from 56 healthy individuals were used, respectively.

The CoV-ETH study launched in May 2020 and included 2,911 voluntary participants from the ETH Zurich community and respective household members, aged 18 to 64 years (**Figure 1**, **Table 1**, **Supplementary Table 1**). The first sampling of blood [at time point 1 (t_1_)] for the collection of plasma and peripheral blood mononuclear cells (PBMC) was in May 2020. The status of respiratory infections prior to the sampling was assessed. Symptoms scores were reported as 0 (without symptoms), 1 (local: with any one or several symptoms, but no fever), and 2 (systemic: fever alone or with any symptom or several). A compound symptoms score was assessed across two screenings (t_1_ and t_2_) by taking the maximum of the two scores for each participant.

**Table 1:** The age distribution, function at ETH Zurich, COVID-19 contact in the last three months prior to sampling, and smoking status of the participants, and number of SARS-CoV-2 tested participants are shown.

	CoV-ETH		Current Study						
			May 2020		Sept. 2020			Total	
	**#**	**%**	**#**	**%**	**#**	**%**		**#**	**%**
Total # participants	2911	100	134	100	134	100			
# surveys answered	2911	100	131	98	120	90	**Function at ETH**		
							Student	37	28
							PhD student	36	27
**Affiliation**							Scientist	15	11
ETH	2106	72	95	71	95	71	Professor	4	3
Household members	804	28	39	29	39	29	Administrative	6	4
**Sex**							Technician	5	4
female	1439	49	69	52	69	52	Other	31	23
male	1470	51	65	48	65	48	**Smoking**		
other	1	0	0	0	0	0	Yes	11	8
**Age range**							No	123	92
18-30	1660	57	91	68	91	68	**Comorbidities**		
31-50	901	31	31	23	31	23	Respiratory	5	4
51-65	349	12	12	9	12	9	High BP	1	1
**Covid tested**							Obesity	1	1
No	2845	98	124	93	90	93	Cardiovascular	0	0
Yes, negative	53	2	1	1	16	1	Diabetes 1	1	1
Yes, positive	8	0	7	5	14	5	Diabetes 2	0	0
–	4	0	2	1	14	1	Immune	1	1
**COVID-19 seroconverted**							HIV	1	1
Yes	113	4	65	49	65	49	Cancer	0	0
**COVID-19 contact before sampling**							Blood cancer	0	0
Not known of	2673	92	98	75	96	80	Kidney	0	0
Probably	125	4	11	8	8	7	Liver	0	0
Yes	107	4	22	17	16	13	Neurological	1	1
**Symptoms before sampling**							Spleen	0	0
No symptoms	1343	46	31	24	55	46	Stroke	0	0
Symptoms, no fever	1239	43	72	55	58	48	None	123	94

In case of seroconversion, determined by seropositivity for RBD using an enzyme-linked immunosorbent assay (ELISA), blood specimens were obtained in September 2020 (at time point 2, t_2_) for plasma and PBMC isolation. At this time, no vaccination was available.

Based on RBD IgG levels, we included 134 probands into our study, of whom 69 were seronegative and 65 were seropositive individuals.

### C+ cohort (positive controls – hospitalized COVID-19 individuals)

The C+ cohort comprised 56 PCR-confirmed SARS-CoV-2 infected samples from 36 unique individuals aged between 18 and 70. The samples were taken between 15 and 152 days after the symptoms’ onset. Blood sample processing was performed as reported earlier (9). Blood collection was performed under institutional review board approval number 2020-039 (ethics committee of the University Medical Center Halle).

### C- cohort (negative controls – pre-pandemic individuals)

Healthy pre-pandemic control samples were collected at the Rockefeller University Hospital, US, between 1996 and 2000 and originated from 56 pre-pandemic healthy individuals aged between 21 and 85. Donor consent for their samples to be used in research was obtained from all participants and the study was approved by the Rockefeller University Ethics Committee. Plasma samples were stored permanently at -80^°^C.

Further information on sample collection and processing can be found in the **Supplementary Methods** section.

### Enzyme-linked immunosorbent assays (ELISA)

All CoV-ETH cohort samples were screened for SARS-CoV-2 specific IgG, IgM, and IgA antibodies targeting the receptor-binding domain (RBD) using a previously described SARS-CoV-2 RBD ELISA (10). Three further in-house immunoassays were developed for the detection of SARS-CoV-2 specific IgG antibodies targeting Spike S1, S2 and Nucleocapsid (N), respectively (**Supplementary Table 1**). To characterize seroconverted participants of the CoV-ETH cohort, C+ and C- cohort samples, six different plasma sample dilutions for each of the individual assays were employed to achieve respective ED50 values. Assay details can be found in the **Supplementary Methods** section.

### T cell analysis

The T cell response assays were performed on PBMCs collected at t_1_ and t_2_ of the CoV-ETH cohort individuals. Each assay plate contained PMBCs collected from a single healthy donor as an intra-assay control (IAC). After overnight cultivation, viability and cell count adjusted to 5 × 10^6^ lymphocytes/mL were assessed by flow cytometry on a MACSQuant^®^ Analyzer 16 (Miltenyi Biotec).

Cells were aliquoted and stimulated with a (1) PBS (negative control), (2) SARS-CoV-2 PepTivator mix (CoV-Mix), (3) Prot_N, (4) Prot_S1, (5) Prot_S, (6) Positive Control and (7) Prot_M. The IACs received only the negative control, positive control and a mix of 10 µM of each human PepTivator CMV pp65, PepTivator EBV Consensus, PepTivator AdV5 Hexon (Miltenyi Biotec) for four hours (**Supplementary Figure 1A, B**). Afterwards, cell staining was performed against CD14-VioBlue^®^ (Miltenyi Biotec, Cat. No.130-110-525), CD20-VioBlue^®^ (Miltenyi Biotec, Cat. No.130-111-531), CD8-VioGreen™ (Miltenyi Biotec, Cat. No.130-110-684), CD4-VioBright™515 (Miltenyi Biotec, Cat. No.130-114-535), IFNγ-PE (Miltenyi Biotec, Cat. No.130-113-496), IL-2-PE-Vio615 (Miltenyi Biotec, Cat. No.130-111-307), TNFα-PE-Vio^®^770 (Miltenyi Biotec, Cat. No.130-120-492), CD3-APC (Miltenyi Biotec, Cat. No. 130-113-135), CD154-APC-Vio^®^770 (Miltenyi Biotec, Cat. No.130-114-130), and cells were analyzed by flow cytometry. Assay details can be found in the **Supplementary Method** section.

Flow data files in MQD format were directly imported to FlowJo™ v10.6 (BD Life Sciences) for the analysis. Singlet viable CD3 T cells, CD8 cytotoxic T cells and CD4 T helper cells were analyzed using quadratic gating for co-expression of TNF and IFN-γ, as well as IL-2 and CD154 (**Supplementary Figure 1B**). Gate thresholds were set based on negative and positive controls of each sample. Cell counts (#; cells per quadrant), frequency of parent (%; portion of cells in a specific quadrant) and mean fluorescence intensity (MFI; per cells in the assigned quadrant) values were reported for the double positive populations and calculated for single positive populations. In total, 155 parameters were reported for each analyzed sample well.

### Neutralizing antibodies

The determination of neutralizing antibody titers (nAb) in all selected samples of the CoV-ETH cohort and the samples of the C+ cohort was performed as reported earlier (9). Fourty samples of the C- cohort as well as pre-pandemic samples reported in the previous study (9) showed negativity.

### Serological data analysis

For the re-sampling of PBMC in September, we defined a raw optical density (OD) threshold for RBD IgG ≥ 0.7 or IgM ≥ 1.0 or IgA ≥ 1.0. To validate the performance of the serological tests, receiver operating characteristic (ROC) curves were constructed from the pre-pandemic C- cohort and the SARS-CoV-2 PCR-confirmed C+ cohort.

### Statistical and machine learning analyses

#### Establishment of antibody level cutoffs

**Figure 2:**
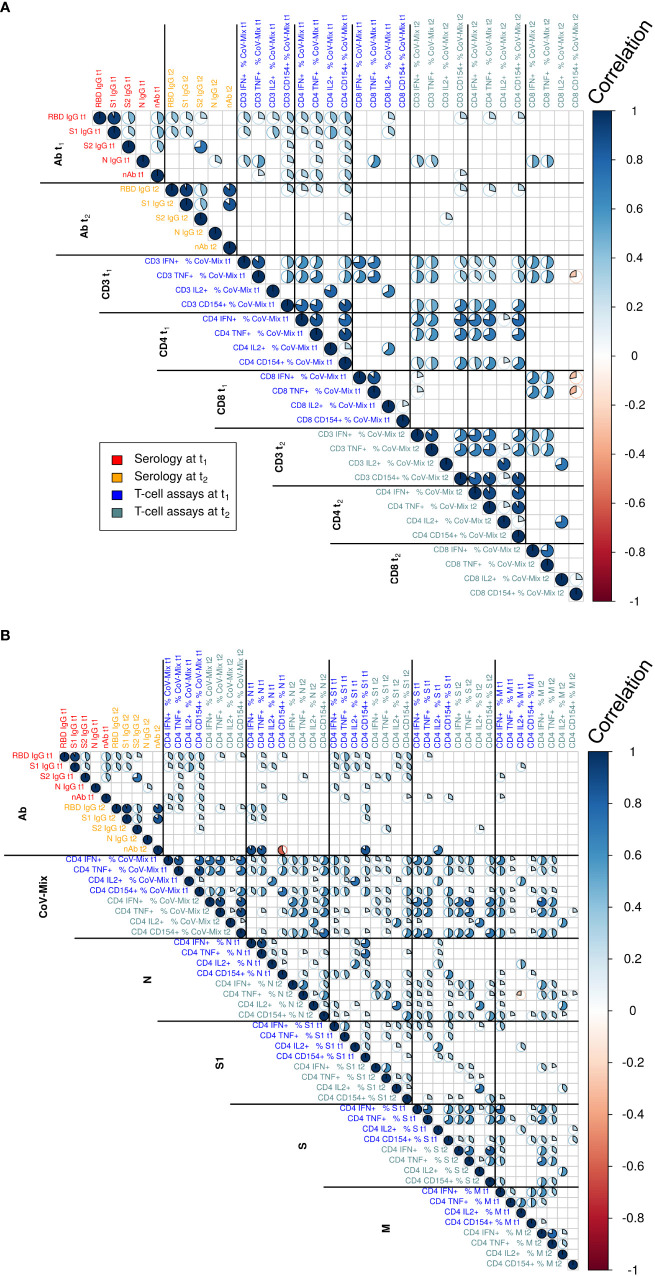
Correlation analyses relating to the assessed humoral and cellular parameters at time points t_1_ and t_2_ relating to **(A)** CD3, CD4 and CD8 T cells and a detailed analysis of **(B)** the different stimulating peptides in CD4 T cells. Text color indicates serology and T cell assay measurements at t_1_ and t_2_. The magnitude of correlation coefficients is indicated by the color bar to the right. Statistically non-significant correlations are not displayed (t-test at significance level *α*=0.05). Correlation test p-values were adjusted for multiple comparisons using the Benjamini-Hochberg method.

Antibody response was defined as a binary variable based on the measurements acquired from the C+ and the C- cohorts. For each antibody targeting different SARS-CoV-2 antigens and domains (RBD, S1, S2, N and nAb), an optimal range of cutoffs maximizing the balanced accuracy (11) was selected. **Supplementary Table 2** reports optimal cutoff intervals and the corresponding balanced accuracies attained on the control cohort, in addition to sensitivities and specificities. ROC curves for all antibody types are plotted in the **Supplementary Figure 2**. In the current analysis, we used a cutoff of 50, 20, 5, 5 and 20 for RBD, S1, S2, N and nAb, respectively. In addition, the compound antibody response (see **Table 2**) was obtained by aggregating responses across several antibodies targeting different SARS-CoV-2 antigens and domains. A subject was labeled positive if they had the ED50 of RBD ≥ 50 and either ED50 of N ≥ 5, ED50 of S1 ≥ 20 or ED50 of S2 ≥ 5 in at least one of the screenings (t_1_ or t_2_). Otherwise, the subject was assigned to the negative group (**Figure 2**). The resulting compound antibody response comprised two balanced categories: negative (n=69) and positive (n=65). The criteria for defining antibody responses described above are summarized in **Table 2**.

**Table 2:** Definitions of the response with respect to different antibody types.

Antibody Response	Definition of Positivity
RBD	ED50 of RBD ≥ 50 at t_1_ **OR** ED50 of RBD ≥ 50 at t_2_
N	ED50 of N ≥ 5 at t_1_ **OR** ED50 of N ≥ 5 at t_2_
S1	ED50 of S1 ≥ 20 at t_1_ **OR** ED50 of S1 ≥ 20 at t_2_
S2	ED50 of S2 ≥ 5 at t_1_ **OR** ED50 of S2 ≥ 5 at t_2_
nAb	ED50 of nAb ≥ 20 at t_1_ **OR** ED50 of nAb ≥ 20 at t_2_
Compound	(ED50 of RBD ≥ 50 at t_1_ **AND** [ED50 of N ≥ 5 at t_1_ **OR** ED50 of S1 ≥ 20 at t_1_ **OR** ED50 of S2 ≥ 5 at t_1_]) **OR** (ED50 of RBD ≥ 50 at t_2_ **AND** [ED50 of N ≥ 5 at t_2_ **OR** ED50 of S1 ≥ 20 at t_2_ **OR** ED50 of S2 ≥ 5 at t_2_])

For RBD, N, S1, S2, and nAb, the subject was defined as positive if ED50 exceeded the specified threshold at either time point t_1_ or t_2_. The compound antibody response was defined by combining criteria based on RBD, N, S1, and S2.

#### Preprocessing

As preprocessing steps before training and validating predictive models, raw T cell counts, frequencies and MFIs were (i) normalized by subtracting the corresponding T cell response to the negative control treatment (*background subtraction*) and (ii) standardized by subtracting the mean and scaling to the unit variance across participants (*standardization*). No further feature transformations were performed. During exploratory analysis, we also considered normalization by dividing by T cell response to the negative control treatment. Results for this normalization technique are reported in **Supplementary Table 3**. Henceforth, all reported results relate to the normalization by subtraction.

#### Statistical and machine learning models

To explore relationships between the compound antibody and T cell responses, we leveraged statistical and ML predictive models. We trained and validated predictive models classifying negative and positive antibody response based on T cell measurements. ML analysis was performed in the Python programming language (version 3.8.8) (12) and in the R programming language (version 4.2.2) (13). We performed binary classification using the (i) logistic regression (LR) (14) as implemented in the scikit-learn library (version 0.24.1) (15), and (ii) gradient boosting (GB) (16), as implemented in the XGBoost library (version 1.3.3) (17). GB was considered in addition to the LR due to its ability to model nonlinear relationships without extensive feature engineering and transformation. No hyperparameter tuning was performed for GB, and default hyperparameter values were used to avoid overfitting. Features, aka predictors or explanatory variables, were given by T cell measurements expressed as (i) #, (ii) %, and (iii) MFIs, or a combination thereof.

#### Model evaluation and comparison

The predictive performance of models was evaluated using stratified bootstrapped train-test split. In this procedure, the dataset was resampled with replacement 1,000 times. For every bootstrap resample, the resampled dataset was split into the train (80%) and test (20%) sets, stratified by the response variable, and a predictive model was trained and tested. Test set performance was aggregated across the bootstrap resamples, and empirical confidence intervals (CI) were constructed. The bootstrapping (18) was performed to construct more conservative confidence intervals and was preferred to repeated train-test splits, standard in the ML literature, since the latter might produce misleading CIs and significance (19). To compare different predictive models, we used areas under the receiver operating characteristic (AUROC) and precision-recall (AUPRC) curves (20) computed on held-out test data. In addition, we evaluated the models’ balanced accuracy (BA) (11), sensitivity, and specificity by considering a threshold of 0.5 on predicted probabilities. The latter evaluation metrics are reported in **Supplementary Table 6**.

## Results

### Descriptive statistics of the cohort and primary serology data

The descriptive statistics of the total number of 2,911 participants of the CoV-ETH study and the subgroup used in this study are shown in **Table 1**. Overall, the cohort is young (50% of participants younger than 30) and healthy (>90% of participants without any underlying cardiovascular risk factors). In May 2020, we found 4% of seropositive cases, which is in line with the reported numbers in Geneva in April 2020 (21). Interestingly, more than 80% of seropositive participants had no prior history of COVID-19 symptoms and therefore are considered asymptomatic, while 20% of individuals exhibited mild to moderate disease symptoms.

We next assessed the antibody titers against RBD, S1, S2 and N in the plasma of 165 donors, including 96 seropositive, and 69 randomly chosen donors displaying antibody levels below all positivity thresholds, and thus considered as seronegative (**Figure 1**). Thirty-one donors that, based on RBD measurements, were initially included in the study, were subsequently excluded because of a false-positive RBD cross-reactive signal. The cross reactivity was identified through a lack of additional seropositivity for S1, S2 or N and an absence of RBD decay over a period of 1 year. Finally, 65 individuals were identified as seroconverted. The details related to the distinct antibodies of the individual participants are shown in **Supplementary Figure 3**.

### Basic exploratory data analysis

T cell responses were assessed by stimulating isolated PBMCs with SARS-CoV-2 protein-derived peptide pools and by determining frequencies of reacting T cells *via* flow cytometry. We (i) evaluated whether flow cytometry read-out alternatives were impacted by normalization strategies accounting for background noise, (ii) assessed the test specificity and (iii) determined the repeatability of the measurements. A considerable proportion of measurements resulted in numerically negative values after normalization (**Supplementary Figure 4**), a procedure that is strongly recommended to account for background noise, particularly in a low-input PBMC setting. We found that the general stimulation of T cells did not lead to a spurious association with humoral antibody status against SARS-CoV-2. Lastly, there was considerable variation for the cytokine assays measuring T cell responsiveness, especially in the negative control stimulation, due to only few cells per target quadrant (**Supplementary Table 4**). Nevertheless, all variances were lower than the differences between the infected and non-infected individuals found later. The detailed analysis can be found in the Further Results Supplementary section.

### B and T cell responses against SARS-CoV-2 proteins

#### Correlation analysis

To assess associations between antibody and T cell responses we initially performed a Pearson’s correlation analysis displayed in **Figure 2**. **Figure 2A** shows correlations among humoral and cellular parameters at time points t_1_ and t_2_ for CD3, CD4 and CD8 T cells. **Figure 2B** provides correlation analysis results for different stimulating peptides of CD4 cells. The analysis of multiple populations of circulating T cells reactive to SARS-CoV-2 revealed specific responses in the total CD3 T cell compartment, as well as the CD4 and CD8 T cell subsets in response to peptide pools covering S1, S2 as well as the N and M proteins. We found a high correlation of frequency of responding CD3 and CD4 T cells against the different respective peptide pools between t_1_ and t_2_, indicating that SARS-CoV-2 induces a stable cellular immune response over four months. Cytokine production of IFN-γ, TNF and CD154 correlated strongly in CD4 T cells within and between t_1_ and t_2_. Stimulation of CD8 T cells resulted in IFN-γ and TNF release, which showed a positive correlation within t_1_ and t_2_ and between these time points. Furthermore, there was a high correlation of IL-2 production between CD3, CD4 and CD8 T cells at t_1_, which largely disappeared at the later time point t_2_, demonstrating that the IL-2 response was short-lived.

When searching for associations between humoral and T cell responses to SARS-CoV-2 infection, we found moderate correlations of RBD and S1 with CD4 T cells for all cytokines at t_1_. However, this correlation was lost at t_2_. A positive correlation of nAb was only detected with IL-2 production in CD3, CD4 and CD8 T cells at t_1_. This association however was also lost at t_2_, most likely due to a faster decay of RBD/S1 antibodies. We observed almost no correlations between CD4 and CD8 T cells, neither between nor across time points t_1_ and t_2_. A greater correlation of CD4 T cells across t_1_ and t_2_ than for CD8 T cells may suggest that CD4 T cells have a longer half-life.

#### General predictive relationships

In the next step, we assessed if a statistical model and ML approach would relate T cell stimulation to antibody status. We modeled the relationship between T cell and compound antibody responses using LR and GB models. **Table 3** summarizes the test-set performance of the predictive models trained on numbers [#], frequencies [%] and mean fluorescence intensity [MFI] data.

**Table 3:** Test-set bootstrapped areas under receiver operating characteristic (AUROC) and precision-recall (AUPRC) curves of logistic regression (LR) and gradient boosting (GB) models predicting the compound antibody response based on T cell data.

Model	AUROC	AUPRC
Rand. guess	0.50	0.50
LR #	0.92; [0.73, 1.00]	0.93; [0.74, 1.00]
LR %	0.91; [0.69, 1.00]	0.92; [0.71, 1.00]
LR MFI	0.87; [0.64, 1.00]	0.88; [0.61, 1.00]
LR #,%	0.95; [0.79, 1.00]	0.95; [0.77, 1.00]
GB #	0.96; [0.80, 1.00]	0.96; [0.81, 1.00]
GB %	0.95; [0.76, 1.00]	0.95; [0.75, 1.00]
GB MFI	0.93; [0.73, 1.00]	0.93; [0.69, 1.00]
GB #,%	0.96; [0.80, 1.00]	0.96; [0.76, 1.00]

Models were trained on different data types, namely, ‘#’ denotes models trained on counts; ‘%’ denotes models trained on percentages; ‘#,%’ denotes models trained on both counts and percentages; and ‘MFI’ denotes models trained on mean fluorescence intensities. AUROCs and AUPRCs are reported as the average over 1,000 bootstrap resamples and a 95% empirical confidence interval. As a naïve baseline, we report the expected AUROC and AUPRC of a random guess.

The data indicate that both LR and GB were able to capture a significant association of T cell counts and frequencies to the antibody response. Note that for all models, the lower bound of the empirical CI was above the expected performance of a random guess. Moreover, the found association was remarkably strong; for instance, the GB model trained on counts had an average test-set AUROC and AUPRC of 0.96 (95% empirical CI [0.80, 1.00]) and 0.96 (95% CI [0.81, 1.00]), respectively. MFI measurements featured a slightly weaker association, resulting in lower average AUROC and AUPRC and wider CIs. Thus, there was an overall significant association between T cell reactivity and antibody titer.

#### Predictors of B cell and T cell association

**Figure 3:**
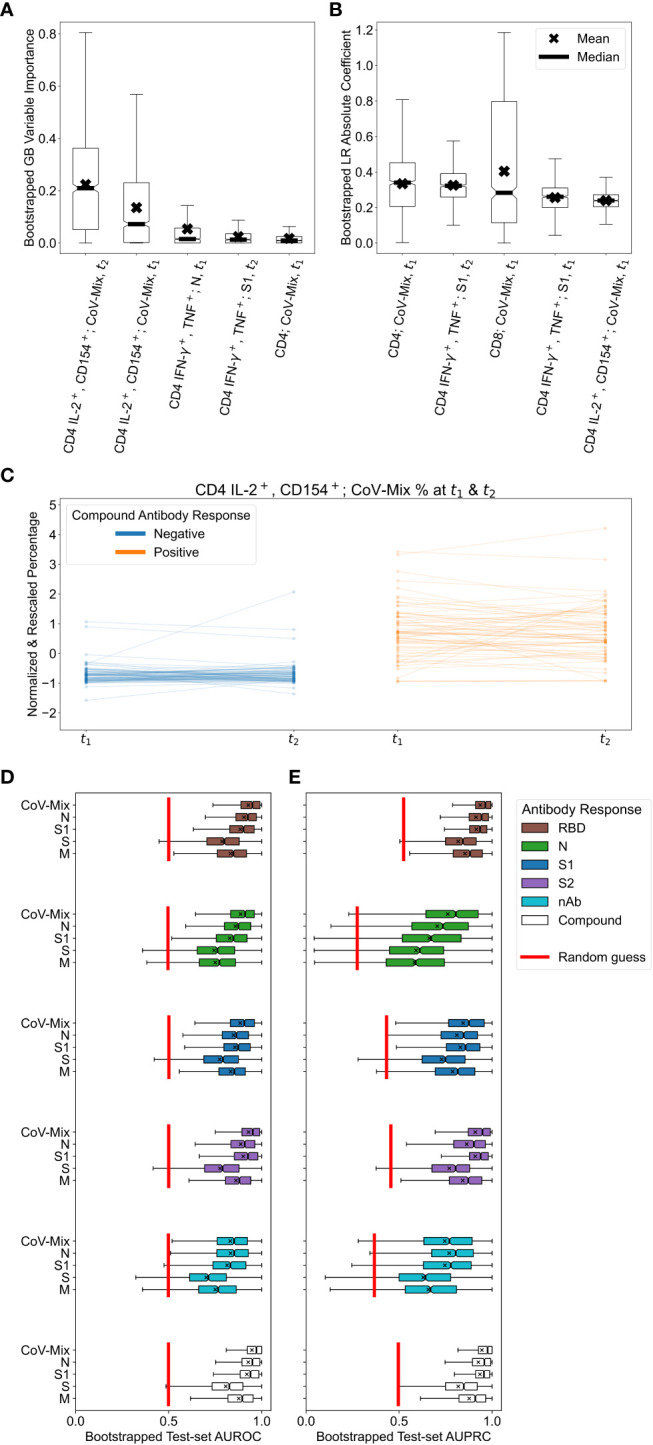
Results of applying logistic regression (LR) and gradient boosting (GB) to relate T cell and antibody responses. **(A, B)** Variable importance values for the top 5 most relevant predictors in the **(A)** GB and **(B)** LR models, trained on T cell percentages. Box plots were obtained by resampling the dataset 1,000 times with replacement. We attribute large variations in importance and coefficient values to the small sample size and correlations among features. **(C)** Changes in the normalized and standardized percentage of CD4 IL-2^+^/CD154^+^ T cells stimulated with CoV-Mix at t_1_ and t_2_. Participants with negative and positive compound antibody responses can be differentiated quite well based on this measurement alone. **(D, E)** Test-set bootstrapped areas under **(D)** receiver operating characteristic (AUROC) and **(E)** precision-recall (AUPRC) curves of GB models predicting various antibody responses based on different treatments. For reference, we plotted the expected performance of a random guess in red.

We next assessed which variables contributed the most to the association between T cell stimulation and antibody status. To this end, we explored the most important predictor variables in LR and GB. In GB, a feature’s importance was quantified by the gain in accuracy from adding the variable of interest to the set of all the other variables. In LR, the conventionally used absolute value of the rescaled coefficient was utilized. **Figures 3A, B** show each model’s top 5 most important variables. For the GB, the most important predictors were the frequencies of CD4 IL-2^+^/CD154^+^ T cells at both time points. The LR model also ranked the percentage of CD4 IFN-γ^+^/TNF^+^ T cells at t_2_ alongside total frequencies of CD4 and CD8 T cells even higher. In conclusion, after stimulation with specific SARS-CoV-2 antigens, multifunctional IL-2^+^/CD154^+^ or IFN-γ^+^/TNF^+^ CD4 T cells clearly reveal a relationship between seropositivity and T cell reactivity.

Given the findings above, we were interested in defining which viral antigens triggered the strongest CD4 T cell response. In addition, we explored if the time-point of sampling led to varying outcomes indicating decay of T cell responsiveness over time. Furthermore we addressed the question if a repeated measurement of T cell stimulation added benefit to the T cell stimulation read-out. **Figure 3C** shows changes in the normalized and standardized percentage of CD4 IL-2^+^/CD154^+^ T cells responding to CoV-Mix (peptide pool mix against Prot_N, Prot_S1, Prot_S) across t_1_ and t_2_. The unstandardized percentages are reported in **Supplementary Figure 6**. Participants without previous SARS-CoV-2 infection tended to have a considerably lower percentage of CoV-Mix-responding CD4 T cells than infected participants. Neither the change across two time points nor the slope of change were associated with the antibody response. In conclusion, CD4 T cells that are dual-positive for IL-2^+^/CD154^+^ and stimulated to CoV-Mix alone can be used to discriminate between healthy and infected individuals. The mix of peptides from all three antigens triggered the strongest CD4 T cell response, followed by S1 and N, which were second most strongly associated with the humoral response. As there was no change in T cell responsiveness across the two time points, only one sampling would have been sufficient to discriminate an infected from a non-infected individual.

#### T cell sublineages

We next assessed which T cell subtype featured the best concordance with T cell response and antibody titer. We trained GB models only on the subsets of % corresponding to CD3, CD4, and CD8 T cell data. **Table 4** reports the test-set performance of these models in terms of AUROC and AUPRC. We found that stimulation of CD4 T cells alone allowed discriminating between healthy and infected individuals, while CD8 T cells did not. Interestingly, we drew a similar conclusion from the sparse principal component analysis on the T cell data (see the **Supplementary Material** and **Supplementary Figure 5**), showing that the first principal component strongly correlated with the antibody response and mainly comprised CD4 T cell measurements.

**Table 4:** Test-set bootstrapped areas under receiver operating characteristic (AUROC) and precision-recall (AUPRC) curves of gradient boosting (GB) models predicting the compound antibody response based on T cell percentages.

Model	AUROC	AUPRC
Rand. guess	0.50	0.50
GB CD3,4,8	0.95; [0.76, 1.00]	0.95; [0.75, 1.00]
GB CD3	0.94; [0.74, 1.00]	0.94; [0.72, 1.00]
GB CD4	0.96; [0.79, 1.00]	0.96; [0.78, 1.00]
GB CD8	0.52; [0.20, 0.83]	0.58; [0.28, 0.87]

Models were trained on different subsets of features, namely, ‘CD3’ denotes the model trained only on CD3 T cell type data; ‘CD4’ denotes the model trained only on CD4 T cell type data; ‘CD8’ denotes the model trained only on CD8 T cell type data; and ‘CD3,4,8’ denotes the model trained on all T cell types. AUROCs and AUPRCs are reported as the average over 1,000 bootstrap resamples and a 95% empirical confidence interval. As a naïve baseline, we report the expected AUROC and AUPRC of a random guess.

#### Specific antigens and antibody types

To investigate which antigens were associated with the strongest correlation of humoral and T cell response, we performed a more detailed analysis evaluating how predictive the separate treatment with peptide pools covering the SARS-CoV-2 S1, S2, M and N were for different antibody responses. **Figures 3D, E** show the corresponding test-set AUROCs and AUPRCs achieved by GB models trained on the T cell frequency.

Considering the six different antibody responses to RBD, S1, S2 and nAb (**Table 2**), we found that GB models that were based on cell responses stimulated with S- and M-peptide pools, tended to have lower average AUROCs and AUPRCs at predicting all of the response types and larger performance variability across bootstrap resamples. These results suggest that the simultaneous presence of antibodies against RBD, S1, S2, and N most strongly correlated with the T cell response. The antibody response against RBD alone resulted in a comparable association. Neither N nor nAb alone gave conclusive evidence. Yet, here the number of positive cases was low, displaying a high variability (in the case of N due to the fast antibody decline between t_1_ and t_2_).

#### Symptoms and T cell reactivity

**Figure 4:**
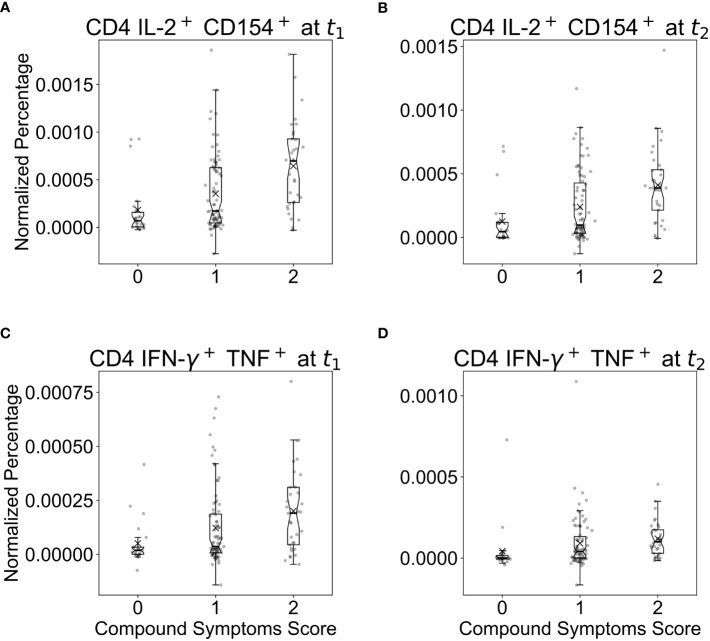
Boxplots of normalized and standardized T cell percentages against the compound symptoms score across two time points (by taking the maximum score) for **(A, B)** CD4 IL-2^+^/CD154^+^ and **(C, D)** CD4 IFN-γ^+^/TNF^+^ T cells, which were previously found to be important predictors of the compound antibody response. Herein, symptoms scores were reported as 0 for subjects without symptoms, 1 for subjects with any one or several symptoms but no fever, and 2 for subjects with fever alone or with any other symptoms. A compound symptoms score was assessed across t_1_ and t_2_ by taking the maximum of the two scores for each participant.

Finally, we assessed if the occurrence of symptoms during SARS-CoV-2 infections correlated with the magnitude of the T cell response, e.g. if individuals with different compound symptoms scores had comparable or different SARS-CoV-2-specific T cell responses. We examined T cell frequencies and self-reported compound symptoms scores across t_1_ and t_2_. **Figure 4** shows normalized T cell frequencies against the compound symptoms score for CD4 IL-2^+^/CD154^+^ and CD4 IFN^+^/TNF-α^+^, which revealed to be most important for the prediction of compound antibody responses (see **Figures 3A, B**). Participants with a compound symptoms score of 2 had a higher average T cell frequency and higher variability than those with a compound symptoms score of 0, particularly for CD4 IL-2^+^/CD154^+^ T cells. We trained and validated a GB model to predict the compound symptoms score based on T cell percentages, however, the model’s test-set AUROC (0.52; 95% CI: [0.27, 0.78]), computed by averaging over all possible one-vs-one pairwise class combinations and balanced accuracy (0.36; 95% CI: [0.17, 0.66]), were not significantly different from the expected performance of a random guess. To conclude, the frequency of dual positive IL-2^+^CD154^+^ CD4 T cells tended to be higher in COVID-19-positive individuals with mild symptoms, and was highest in individuals with fever. However, our moderately-sized dataset falls short of providing evidence for a significant association between T cell response and symptoms score.

## Discussion

Supervised machine learning approaches have been gaining increased attention in many application domains, including immunology (22) and the analysis of COVID-19 data (23). They can be easily applied to large high-dimensional datasets and could help discover predictive patterns and associations among measured covariates and response variables. This approach is especially helpful in studying the complex interactions of antibodies and T cell subsets during an immune response. In addition, it can inform regarding differences in the kinetics between the different arms of the immune response. Uncovering such interactions could facilitate the optimization of vaccines and the identification of potentially critical COVID-19 cases, which require special and time-sensitive medical care. The current study exemplified the use and benefit of ML techniques to describe complex, nonlinear, and nonadditive relationships between humoral and T cell responses.

The major results of our correlation analysis on the antibody levels are in line with previous findings on COVID-19 immune responses, as we found that antibodies targeting the S1-subunit of the SARS-CoV-2 spike protein, which exhibits a high mutation rate and mediates the binding to the receptor on the surface of target cells, make up the largest fraction of nAbs (24). Antibodies targeting the S2-subunit, which has a relatively low tolerance for sequence variation and mediates viral cell membrane fusion, only contributes a comparably small fraction of nAb (25).

Correlation analysis of the measured soluble and cellular responses revealed a yet underestimated role of T lymphocytes, especially of CD4 T helper cells to predict antibody titers. It confirmed previous reports suggesting SARS-CoV-2 specific antibodies decline faster than SARS-CoV-2 reactive T cells, which showed a much longer persistence (26–28). However, our analysis revealed a strong correlation between antibody and CD4 T cell responses. Thus, solely based on the strength of the CD4 T cell response, it was possible to identify individuals who mounted high SARS-CoV-2 antibody titers. The identification of such correlations might depend on the underlying antigen but could nevertheless prove valuable for the improvement or development of novel vaccines, to reach higher antibody titers and reduce vaccination failure rates. Moreover, our CoV-ETH study consisted of a young study population that lacked severe cases. SARS-CoV-2-reactive CD4 T cells predisposing to favorable disease courses have been described in young and unexposed individuals, but with declining numbers in risk groups (29–31). Generally, CD4 T cells are critical for the activation and maturation of B cells into antibody-producing plasma cells. Since CD4 memory T cells are generated after infection and vaccination, they are considered beneficial to mount a faster antibody response upon reinfection. As we show almost no decline in antigen-specific T cells within the study period, future vaccines might employ techniques to induce a long-lasting CD4 memory T cell compartment. This could be instrumental in providing immune protection *via* fast stimulation of SARS-CoV-2 nAb production. However, such approaches would require determining the extent of sufficient protection from infection *via* a memory CD4 T cell compartment, when antibodies are no longer detectable and when antibody epitopes have undergone mutations.

Of note, the antigen-specific CD8 T cell responses did not correlate with CD4 T cell responses. This might be partly due to the different decay rates of the two T cell subsets, suggesting a faster turnover of antigen-specific CD8 T cells. However, CD4 and CD8 T cell responses have been previously found to correlate with the severity of the disease in a different fashion. While a CD8 T cell response is more prominent in mild courses, a CD4 T cell response is dominant in more severe disease courses, as previously reported (32, 33). We detected a higher percentage of antigen reactive CD4 T cells in individuals that recovered from COVID-19 with mild local symptoms as compared to asymptomatic disease courses, and highest in individuals with fever. Since the CoV-ETH study group did not include severely ill or ICU patients, the role for CD4 T cells in individuals with severe COVID-19 outcomes could not be established. Additional data are therefore required to address the functional role for CD8 T cells, especially since these might also be a target of future vaccines.

Given the presented correlation of CoV-Mix-, S1-, and N-induced T cell stimulation and antibodies, our analysis indicates the feasibility of developing a model that can predict structures of the SARS-CoV-2 proteome and that could be considered as future vaccine targets. Furthermore, our analysis may be useful beyond the scope of this specific research question, as it showcases a machine-learning-based analysis pipeline for immunological data and may interest domain experts seeking to enrich their data analysis toolset. To facilitate this, we made our data analysis readily replicable by publishing the deidentified data and the code (available at https://github.com/i6092467/t-cells-response-sars-cov-2).

Taken together, by applying machine learning we suggest T cells might play a substantial yet underestimated role in the virus-specific immune response. T cell responses might bear the potential of improving future vaccine development, as antibody responses alone are insufficient to provide long-lasting protection (27).

### Limitations

This study does not provide information on acute viral diagnostic in individuals with a previous SARS-CoV-2 infection. No seronegative individuals were included in the study, as these were not screened for within the study design. From the statistical and machine learning perspective, the sample size of 134 subjects is small, particularly given the large number of conducted model comparisons and statistical correlation tests. Therefore, the reported findings are exploratory and need to be interpreted cautiously. A larger cohort would allow corroborating reported results and facilitate using potentially more powerful models, such as neural networks. It would be helpful to validate the resulting ML models and findings on the external data obtained under a similar experimental setup but from a more diverse set of individuals.

## Data availability statement

The original contributions presented in the study are included in the article/**Supplementary Materials**. The data and code are available in a GitHub repository at https://github.com/i6092467/t-cells-response-sars-cov-2. Further inquiries can be directed to the corresponding authors.

## Ethics statement

The studies involving human participants were reviewed and approved by the Cantonal Ethics Commission Zurich (BASEC-Nr. 2020-00949). The patients/participants provided their written informed consent to participate in this study.

## Author contributions

RM, PS, and A-KH organized and performed the sampling, data assessment and analyses and wrote the first draft of the manuscript. CD and MSc interpreted the data and wrote the manuscript. PS, KC, SG, AG, LH, PH, HK, ML, JM, AT, XS, A-KH, NB, SB, LD, MS-d-J, MSa, GS, SZU, TU, and TW performed the blood sampling. PS, KC, SG, AG, ML, JM, and AT performed the cell assays. DS, FA-Q, and SY set up the data pipeline. MB and CS provided the samples of the positive control cohort. CZ and CK set up, organized and performed the clinical blood sampling procedure. JG and MS set up, organized and performed the clinical blood sampling and provided medical care. JG was responsible for the ethical approval. LP and FS performed data assessment, analysis and interpretation. SC performed the neutralizing antibody analysis. JV supervised the ML data analyses. MS and SU conceptualized the study, performed data analyses and interpretation and wrote the manuscript. All authors contributed to the article and approved the submitted version.
