# Supplementary material for: Machine learning analysis of humoral and cellular responses to SARS-CoV-2 infection in young adults
Source: Front Immunol. 2023 May 29;14:1158905. doi: 10.3389/fimmu.2023.1158905 (PMC10258347; doi:10.3389/fimmu.2023.1158905)
Supplement: Supplementary file 1 [file DataSheet_1.pdf]

Supplementary Material: Machine learning analysis of humoral and cellular responses to SARS-CoV-2 infection in young adults

Ricards Marcinkevics, Pamuditha N. Silva, Anna-Katharina Hankele et al.

Material and Methods

CoV-ETH cohort

Study approval

The ethical approval for the CoV-ETH study (CoV-ETH cohort) was obtained from the Cantonal Ethics Commission Zurich (BASEC-Nr. 2020-00949). Written informed consent was received from all participants. The study has been performed in accordance with the Declaration of Helsinki of 1975.

Participants and samples

The CoV-ETH study launched in May 2020 comprising 2,911 voluntary participants of the ETH Zurich community and respective household members, aged 18-64 years (Figure 1, Table 1 and Supplementary Table 2). The first sampling of blood [at time point 1 (t_1)] for the collection of plasma and peripheral blood mononuclear cells (PBMC) was performed between the first release from assigned self-isolation in Switzerland (April 27, 2020) and the opening of schools (May 11, 2020) following a period of controlled self-isolation since March 17, 2020. Individuals with underlying risk factors, specifically respiratory, metabolic as well as cardiovascular illnesses, were included. Exclusion criteria included individuals with active COVID-19 or other respiratory tract infection, prolonged steroid, chemotherapy or immunosuppressive therapy, cancer treatment or severe autoimmune disease. Baseline factors including age, sex, smoking and previous vaccinations against seasonal flus were documented in an electronic questionnaire. The status of respiratory infections prior to the sampling was likewise assessed. Symptoms scores are reported as 0 (without symptoms), 1 (local: with any one or several symptoms, but no fever), and 2 (systemic: fever alone or with any symptom or several). A compound symptoms score was assessed across two screenings (t_1 and t_2) by taking the maximum of the two scores for each participant.

In case of seroconversion determined by targeting the RBD of spike S1 using an enzyme-linked immunosorbent assay (ELISA), a blood donation to re-collect plasma and PBMC was re-assessed in September 2020 (at t_2).

Sample collection

Human blood samples of the CoV-ETH study were collected either in BD CPT^{TM} tubes or in BD Vacutainer Citrate Tubes (6.0 ml). The latter was centrifuged for 15 minutes at 1500 g at 20 °C. CPT tubes were centrifuged for 20 minutes at 1650 g at 20 °C. In both cases, plasma was transferred into deep well plates and stored at -80 °C until further analysis. The PBMC layer of the CPT tubes was transferred to a washing tube and washed twice with PBS. After washing, the PBMC pellet was resuspended 0.95 ml FBS/DMSO (90/10; v/v), transferred to a cryovial and frozen using a Nalgene MR Frosty (Thermo Fisher Scientific, Walthalm, MA, USA). PBMC were stored in liquid nitrogen until further usage.

<u>C+ cohort (positive controls – hospitalized COVID-19 cases)</u>

Study approval

Blood collection was performed under institutional review board approval number 2020-039 (ethics committee of the University Medical Center Halle).

Participants and samples

The C+ cohort comprised 56 PCR-confirmed SARS-CoV-2 infected samples from 36 unique individuals aged between 18 and 70 that were taken between 15 and 152 days after symptoms onset.

Sample collection

Blood sampling processing was performed as reported earlier (Schultheiß et al., 2020).

<u>C- cohort (negative controls – pre-pandemic samples)</u>

Study approval

Healthy pre-pandemic control samples were collected at the Rockefeller University Hospital between 1996 and 2000. Donor consent for their samples to be used in research was obtained from all participants, and the study was approved by the Rockefeller University Ethics Committee. Plasma samples were stored permanently at -80˚C.

Participants and samples

Plasma samples of the C- cohort originated from 56 pre-pandemic healthy individuals aged between 21 and 85.

Sample collection

Whole blood was collected via phlebotomy in acid citrate dextrose (ACD) or ethylenediaminetetraacetic acid (EDTA) tubes and processed for serum and plasma isolation. Samples were stored at –80°C within 2 h after blood drawing.

Enzyme-linked immunosorbent assay (ELISA)

All CoV-ETH cohort samples were screened for SARS-CoV-2 specific IgG, IgM, and IgA antibodies targeting the receptor-binding domain (RBD) using a previously described SARS-CoV-2 RBD ELISA (Piccoli et al., 2020). Three further in-house immunoassays were developed for the detection of SARS-CoV-2 specific IgG antibodies targeting Spike S1, S2 and nucleocapsid (N), respectively (Supplementary Table 1). Briefly, Greiner high binding 384 well plates were coated with either antigen (Sino Biological, Beijing, China). Spike S1 and Spike S2 assay plates were blocked with 1% BSA. N assay plates were blocked with 0.1% BSA. Plasma samples were diluted (1:100) in assay buffer. SARS-CoV-2 specific IgGs were visualized using an anti-human IgG horse-reddish peroxidase (HRP) and 3,3'5,5'-Tetramethylbenzidine (TMB) as a substrate in the Spike S2 and N assay. In the Spike S1 assay, an anti-human IgG labeled with biotin was used. For visualization, streptavidin-poly-HRP and TMB as a substrate were added. In all three assays, the reaction was stopped with 2 M H_2SO_4. Plate reading was performed on a BioTek cytation 3 imaging reader at 450 nm (BioTek Instruments, Vermont, USA). All assay-specific parameters are listed in Supplementary Table 1.

For further characterization of all seroconverted samples, of all C+ as well as of all C- cohort samples, six different plasma sample dilutions for each of the individual assays were employed to achieve respective ED50 values.

T cell analysis

Sample description and arrangement

The T cell response assays were performed on PBMCs collected at two time points from 134 donors. These samples were arranged in such a way that 22 samples representing 11 donors at two time points were distributed over two 96-well plates each. Each assay plate contained PMBCs collected from a single healthy donor as an intra-assay control (IAC). Per daily batch, two sets of two 96-well plates were processed 2 h 45 min apart to accommodate procedures performed either manually or with the AssistPlus automated pipetting system equipped with the Voyager 300 (volumes greater than 10 µL) or Voyager 12.5 (volumes less than 10 µL) multichannel pipettes. All samples were processed over the course of two weeks.

Cell recovery

Frozen PBMC samples were first recovered in thawing media composed of RPMI base media, 2 mM L-Glutamine and 5% human serum AB with 5 U/mL Benzonase at 37°C. Following washing step, cells were resuspended in either 500 µL, 1 mL or 2 mL of cultivation media (i.e. thawing media without Benzonase) if the cell pellet was small, medium or large, respectively. The 500 µL cultures were seeded in 24-well, the 1 mL or 2 mL cultures in 12-well plates. Cells were cultivated for 12–16 h at 37°C in 5% CO_2 and 95% relative humidity.

Reagent preparation

The Negative Control was prepared as a 10% DMSO in water solution while the Positive Control was CytoStim™, human (Miltenyi Biotec) prepared as a 3X solution in cultivation media. Individual SARS-CoV-2 PepTivator® Peptide Pools (Miltenyi Biotec) Prot_N, Prot_S1, Prot_S and Prot_M were prepared as 10 µM working stocks in 10% DMSO while the PepTivator mix (CoV-Mix) included 10 µM each of all PepTivators except Prot_M. The peptide pools consist of lyophilized peptides, consisting mainly of 15-mer consecutive peptides with 11 amino acids overlap, covering the complete sequence of the respective protein.

T cell assay plate preparation and stimulation

After overnight cultivation, cell count and viability and absolute cell counts were assessed by flow cytometry using 7-AAD (7AAD staining solution, Miltenyi Biotec) on a MACSQuant® Analyzer 16 (Miltenyi Biotec). Concentrations were adjusted to 5×10^6 lymphocytes/mL in an appropriate volume of cultivation media. Samples with enough lymphocytes for all treatment conditions were distributed as 100 µL into their designated wells of the U-bottom 96-well plate using an Assist Plus with the Voyager 300 pipette. Cell suspensions were mixed 4 times before dispensing and excess liquid on the tip was pressed against the side of the well. All samples in volumes less than 720 µL were pipetted manually according to a priority list in the following descending order: (1) Negative Control, (2) SARS-CoV-2 PepTivator mix, (3) Prot_N, (4) Prot_S1, (5) Prot_S, (6) Positive Control and (7) Prot_M. The IACs received only the negative control, positive control and a mix of 10 µM of each human PepTivator CMV pp65, PepTivator EBV Consensus, PepTivator AdV5 Hexon (Miltenyi Biotec) (Supplementary Figure 1a). Immediately after seeding cells, the Voyager 12.5 pipette with the Assist Plus was used to dispense 6 µL of stimulants into the wells of rows A-G as per the priority list with a tip change between dispensing steps. Contents of the wells were manually mixed with a multichannel pipette. After 2 hours at 37°C in a 5 % CO_2 incubator, Brefeldin A (Sigma-Aldrich?), at a final concentration of 2 µg/mL, was added to each well using the Assist Plus with the Voyager 12.5 multichannel pipette following the same parameters used to dispense the stimulants. The plate was incubated for an additional four hours (Supplementary Figure 1b).

Intra- and extracellular staining

Staining solutions were prepared fresh prior to use. A 1X solution of Viability™ Fixable Dye 405/452 was prepared in 1x PBS while a master mix of the following conjugated antibodies was combined in equal parts in Inside Perm buffer (Inside Stain Kit, Miltenyi Biotec) at a 1 to 50 dilution: CD14-VioBlue®, CD20-VioBlue®, CD8-VioGreen™, CD4-VioBright™515, IFNɣ-PE, IL-2-PE-Vio615, TNFα-PE-Vio®770, CD3-APC, CD154-APC-Vio®770 (Miltenyi Biotec).

The following steps involving the transfer, aspiration and dispensing of cell suspensions and reagents were performed using semi-automated programs controlling the Assist Plus equipped with the Voyager 300 pipette and manual mixing with a separate multichannel pipette. Cells were first diluted with 100 µL autoMACS® Pro (aMP) running buffer, mixed and transferred to a V-bottom 96-well plate with tip changes between transfers before being centrifuged at 400 × g for 5 min at RT. Wells were aspirated and replaced with 100 µL of Viability™ solution and manually mixed with a multichannel pipette by pipetting up and down 4x with half the volume. Cells were incubated for 10 min at RT in the dark. Afterwards, 100 µL aMP running buffer was added to each well, the plate was centrifuged and supernatant was aspirated. One-hundred microliters of aMP buffer was dispensed and cells were resuspended in this buffer manually. Cells were then fixed by adding 100 µL Inside Fix (Inside Stain Kit, Miltenyi Biotec) and incubated for 20 min at RT in the dark. The plate was centrifuged, supernatant removed and cells were resuspended in Inside Perm buffer in a similar manner. Plates were then centrifuged and supernatants were replaced this time with the 100 µL staining mix and cells were manually resuspended. After incubating for 10 min at RT in the dark, 100 µL of Inside Perm was dispensed into the wells before the plate was centrifuged. The supernatant was completely aspirated before 200 µL of aMP buffer was added and cells resuspended manually. Cell suspensions were transferred to a fresh U-bottom 96-well plate and kept at 4°C prior to acquisition on two MACSQuant® Analyzer 16 devices equipped with robotic needle arm, MACS® MiniSampler Plus automated 96-well plate handler and MACSQuant® Buffer Supply Station 20L (Miltenyi Biotec). Devices were calibrated daily and device-specific compensation was applied during acquisition. All samples were analyzed during a period of two weeks.

Further Results

<u>Basic Exploratory Data Analysis</u>

The flow cytometric readout was assessed as count, percentage and MFI. For each sample, a negative control without any stimulatory peptide was applied to account for background noise. The normalization accounts for the background noise and can be done by either determining the difference or the quotient

between positive cells and the negative control. We therefore wanted to initially assess if normalization significantly impacted either #, % or MFI.

We examined the distributions of observed #, % and MFIs across different assay readouts (TNF, IFN-γ, IL-2, and CD154) before and after normalization by background subtraction. Supplementary Figure 4 depicts the empirical cumulative distribution functions (eCDF) for (a) #, (b) % and (c) MFIs, for the four assays. We observed via IL-2 as a readout, for antigen reactive T cells, much higher median raw # and % and much lower median raw MFI. Expectedly, the background subtraction (dotted eCDFs correspond to normalized measurements) introduced many negative entries, in particular, 38, 37, 51, and 38% of the normalized T cell # were negative for TNF, IFN-γ, IL-2 and CD154 as readout parameters, respectively. The # measurements <20 might be considered unreliable. Upon normalization, many # were very low, in particular, 66, 75, 63, and 77% of all normalized # were < 20 for IFN-γ, TNF, IL-2, and CD154. Note that we did not omit or mask low or negative normalized measurements.

Since the T cell measurements included negative and positive control treatments, as a sanity check, we assessed the association between measurements for these treatments and the compound antibody response. Ideally, we expected a GB model to be unable to predict the response better than a random guess based on control measurements alone. A random guess is expected to have an AUROC and AUPRC of 0.50. A GB model trained on positive controls, achieved an average AUROC and AUPRC of 0.51 (empirical 95% CI: [0.19, 0.80]) and 0.57 (95% CI: [0.27, 0.87]), respectively. For the negative controls, the model had an AUROC of 0.47 (95% CI: [0.17, 0.78]) and AUPRC of 0.54 (95 % CI: [0.23, 0.86]). In both cases, the CIs were very wide and contained the expected performance of a random guess, suggesting no significant association between control treatment measurements and the compound antibody response. Taken together, we did not find any association of our stimulation control of viral proteins from other pathogens with SARS-CoV-2 antibody status. We therefore concluded the lack of non-specific T cell responsiveness and the validity of the ML approach not to predict nonsense associations.

To specifically account for asymptomatic infections with potentially low T cell responses, we finally determined the repeatability of the measurements to appraise the magnitude of the differences in T cell responses observed. The repeatability of measurements was assessed by the coefficient of variation (CV). For each participant, negative and positive controls were measured at each of the two screenings. Although these are not strict technical replicates, ideally, we expected low variability in the repeated measurements. The CV was computed based on participants with a negative compound antibody response. For TNF, IFN-γ, and IL-2 the CV was at approximately 30-40%, whereas CD154 measurements had CVs of 40-50 %. Exact CVs are reported in Supplementary Table 4. On average,

for all assays, the CV was higher than 30% observed for RBD measurements. In general, this analysis suggests considerable variability in measurements, which might pose a challenge when predicting the antibody response based on T cells, particularly if the responses are not strong. The findings are as expected for rare cell analyses. Unfortunately, no alternative assessments were available for assays with low cell numbers.

The results were in line with our further assessment of intra-assay repeatability on 30 repeated control measurements performed on a single donor. For the negative control treatment, all assays expectedly had high CVs: 40-50% for TNF and IFN, 55% for IL-2, and 85% for CD154. For the other two treatments, CVs were more comparable to the results reported before: TNF and IFN-γ assay measurements had CVs around 20-25%, IL-2 and CD154 had 30-40%. Detailed results are reported in Supplementary Table 5.

Principal Component Analysis

To explore the variability in T cell response measurements, we employed principal component analysis (PCA) and sparse PCA (Johnstone & Lu, 2009), which discovers components composed of few covariates. We examined associations between discovered principal components (PC) and the compound antibody response. Supplementary Figure 5 contains scatter plots of the first two PCs for the (a) normal and (b) sparse PCA.

In both cases, naïve and SARS-CoV-2 infected participants form two clearly distinguishable clusters (multivariate differences between the two groups were statistically significant according to the Hotelling's T^2 test, $\alpha = 0.05$). This suggests an association between T cell and antibody responses. In the sparse PCA, the first component is particularly strongly associated with the antibody response: interestingly, this component is almost solely composed of CD3 and CD4 T cell type measurements; whereas the second PC corresponds to CD3 and CD8 T cells.

Reference

Johnstone IM, Lu AY. On consistency and sparsity for principal components analysis in high dimensions. J Am Stat Assoc (2009) 104:682–93. doi: 10.1198/jasa.2009.0121

Supplementary Figure and Table Legends

Supplementary Figure 1: **(a)** T cell assay plate layout. Two pairs of 96-well plates were successively prepared for analysis per day. Each plate pair contains two time points from 11 subjects in columns 1 to 11 and one IAC sample per plate in column 12. Treatment conditions for subject samples in rows A-G were in descending order: Negative Control (DMSO), SARS-CoV-2 PepTivator mix, Prot_N PepTivator, Prot_S1, Prot_S, Prot_M and Positive Control (CytoStim™, human). Automatic sample acquisition was carried out descending per columns and row H contained assay buffer as a washing well to ensusure no carry over to the next column. The IAC in column 12 row B was stimulated with a mix of PepTivator -CMV pp65, -EBV Consensus and -AdV5 Hexon. **(b)** PBMC preparation for T cell assay. Thawed PBMCs were left in culture to recover overnight, $5x10^5$ cells were plated in wells of round-bottom 96-well plates and stimulants were added for each condition. After 2 hours at 37ºC, Brefeldin A was added and cells were incubated for 4 hours. Next, cells were stained with the live/dead marker Viobility 405/452 Fixable Dye, fixed, permeabilized and stained with the antibody cocktail and analyzed using a MACSQuant Analyzer 16. **(c)** Representative gating strategy depicting a SARS-CoV-2 PepTivator mix stimulated subject sample. The first six plots depict sequential gates; time (excluding bubbles), lymphocytes, singlets 1, singlets 2, viable CD3 T cells and daughter gates with CD4 and CD8 T cell populations. Activation marker and cytokine gates are depicted in the lower six plots; IL-2 vs CD154 (left column) as well as TNF vs IFN-γ (right column) plots from the total viable CD3 T cell (top plot pair), CD4 T cell (middle pair) and CD8 T cell (bottom pair) populations. The activation gates for each sample were set based on each sample's negative control condition and applied to the other treatment conditions of that sample.

Supplementary Figure 2: Receiver operating characteristic (ROC) curves for serological positivity. Curves were obtained from a range of thresholds on **(a)** RBD IgG, **(b)** N IgG, **(c)** S1 IgG, **(d)** S2 IgG, and **(e)** nAb antibody levels. Gray labels display a few of the considered cutoffs. The gray dashed line corresponds to the ROC curve of the random guess. The plots were generated based on the cohort consisting of the PCR-positive (C+) and pre-pandemic negative (C-) controls.

Supplementary Figure 3: The numbers of probands exhibiting positive response w.r.t. different antibody types. For instance, there were 62 probands negative w.r.t. all antibody types, four probands that were positive *only* w.r.t. the compound response, RBD and S2 but not the other antibodies, etc. For a detailed explanation on how to read this plot, see https://upset.app/.

Supplementary Figure 4: Empirical cumulative density functions (eCDF) for T cell **(a)** #, **(b)** % and **(c)** MFIs across TNF, IFN-γ, IL-2, and CD154 assays before and after normalizing by background

subtraction (measurements from all participants were included). Dashed eCDFs correspond to normalized measurements. Raw # and % observed for IL-2 are on average considerably lower than for other assays. Expectedly, normalization introduces many negative values. Thus, for all assays, we observed a significant correlation between # and % before and after normalization.

Supplementary Figure 5: First two principal components (PC) of the T cell data. Each point corresponds to a single participant. Point colors and markers correspond to participants' compound antibody response. **(a)** In the normal principal component analysis (PCA), participants with positive and negative responses form separate, distinguishable clusters. **(b)** Sparse PCA seeks to identify PCs with sparse loadings, i.e. PCs composed of few explanatory variables. In sparse PCA, differences between negative and positive groups are also apparent. Several outliers were omitted from the plots, for the sake of readability. Multivariate differences between the groups are statistically significant (PCA: unpaired Hotelling's T-squared test $p < 0.001, T^2 = 59.54$, 2 and 131 degrees of freedom; sparse PCA: unpaired Hotelling's T-squared test $p < 0.001, T^2 = 37.88$, 2 and 131 degrees of freedom).

Supplementary Figure 6: Changes in the normalized, i.e., background-subtracted, percentage of CD4 IL-2+/CD154+ T cells stimulated with CoV-Mix at t_1 and t_2. Participants with negative and positive compound antibody responses can be differentiated quite well based on this measurement alone. Note that in this plot, percentages were not rescaled to zero mean and unit variance.

Supplementary Table 1: ELISA parameters for SARS-CoV-2-Spike S1 IgG, SARS-CoV-2-Spike S2 IgG and SARS-CoV-2 N IgG ELISA.

Supplementary Table 2: Optimal antibody level cutoffs for predicting PCR test outcome in the cohort consisting of the PCR-positive and pre-pandemic negative controls. For each antibody type, we chose an interval maximizing the balanced accuracy, given by the arithmetic average of specificity and sensitivity. In addition to intervals for the cutoff, we report a range for the balanced accuracy achieved and the number of subjects used to define and evaluate all cutoffs.

Supplementary Table 3: Test-set bootstrapped areas under receiver operating characteristic (AUROC) and precision-recall (AUPRC) curves of logistic regression (LR) and gradient boosting (GB) models predicting the compound antibody response based on T cell data. Two normalization procedures were considered: (i) subtracting the count/percentage observed for the negative control treatment (denoted by '[-]') and (ii) dividing by the count/percentage observed for the negative control treatment (denoted by '[/]'). Models were trained on different data types, namely, '#' denotes models trained on counts; '%' denotes models trained on percentages; '#,%' denotes models trained on both counts and percentages; and 'MFI' denotes models trained on mean fluorescence intensities. AUROCs and

AUPRCs are reported as the average over 1,000 bootstrap resamples and a 95% empirical confidence interval. As a naïve baseline, we report the expected AUROC and AUPRC of a random guess. As can be seen, models trained on the data normalized by background division ('[/]') tended to have lower average AUROCs and AUPRCs and wider CIs.

Supplementary Table 4: Coefficients of variation (CV) for different T cell assay (TNF, IFN-γ, IL-2, and CD154) measurements for negative (neg.) and positive (pos.) control treatments. CVs were assessed by comparing measurements at t_1 and t_2. We evaluated CVs on measurements acquired from all subjects and from the negative subjects only.

Supplementary Table 5: Coefficients of variation (CV) for different T cell assay (TNF, IFN-γ, IL-2, and CD154) measurements for three different treatments. CVs were computed based on 30 repeated measurements performed on a single donor.

Supplementary Table 6: Test-set bootstrapped balanced accuracies (BA), sensitivities, and specificities (spec.) for logistic regression (LR) and gradient boosting (GB) models predicting the compound antibody response based on T cell data. A threshold of 0.5 was used to predict binary classes. Models were trained on different data types, namely, '#' denotes models trained on counts; '%' denotes models trained on percentages; '#, %' denotes models trained on both counts and percentages; and 'MFI' denotes models trained on mean fluorescence intensities. Metrics are reported as the average over 1,000 bootstrap resamples and a 95% empirical confidence interval. As a naïve baseline, we report the expected performance of a random guess.

Supplementary Figures and Tables

Supplementary Figure 1

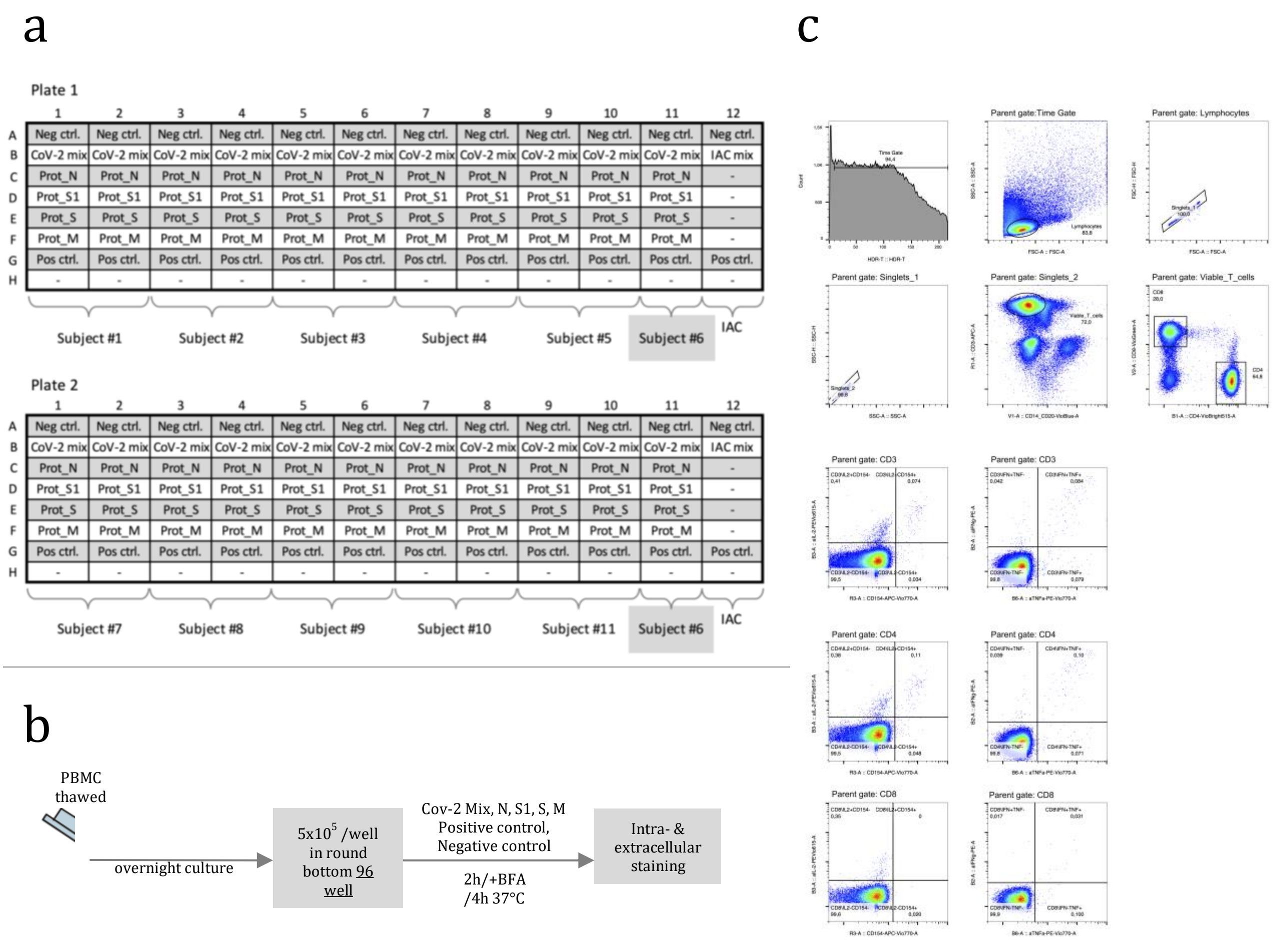

Supplementary Figure 2

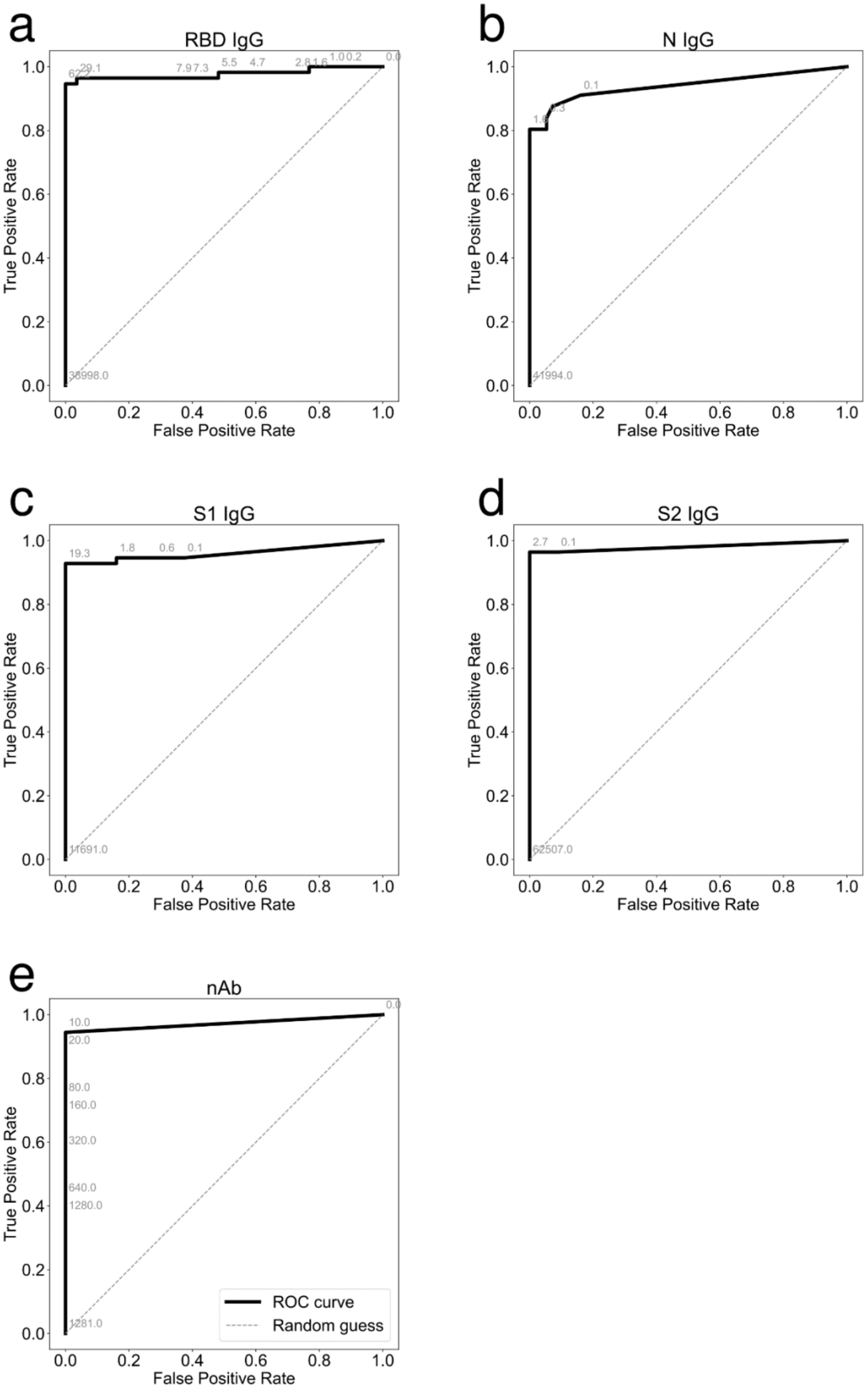

Supplementary Figure 3

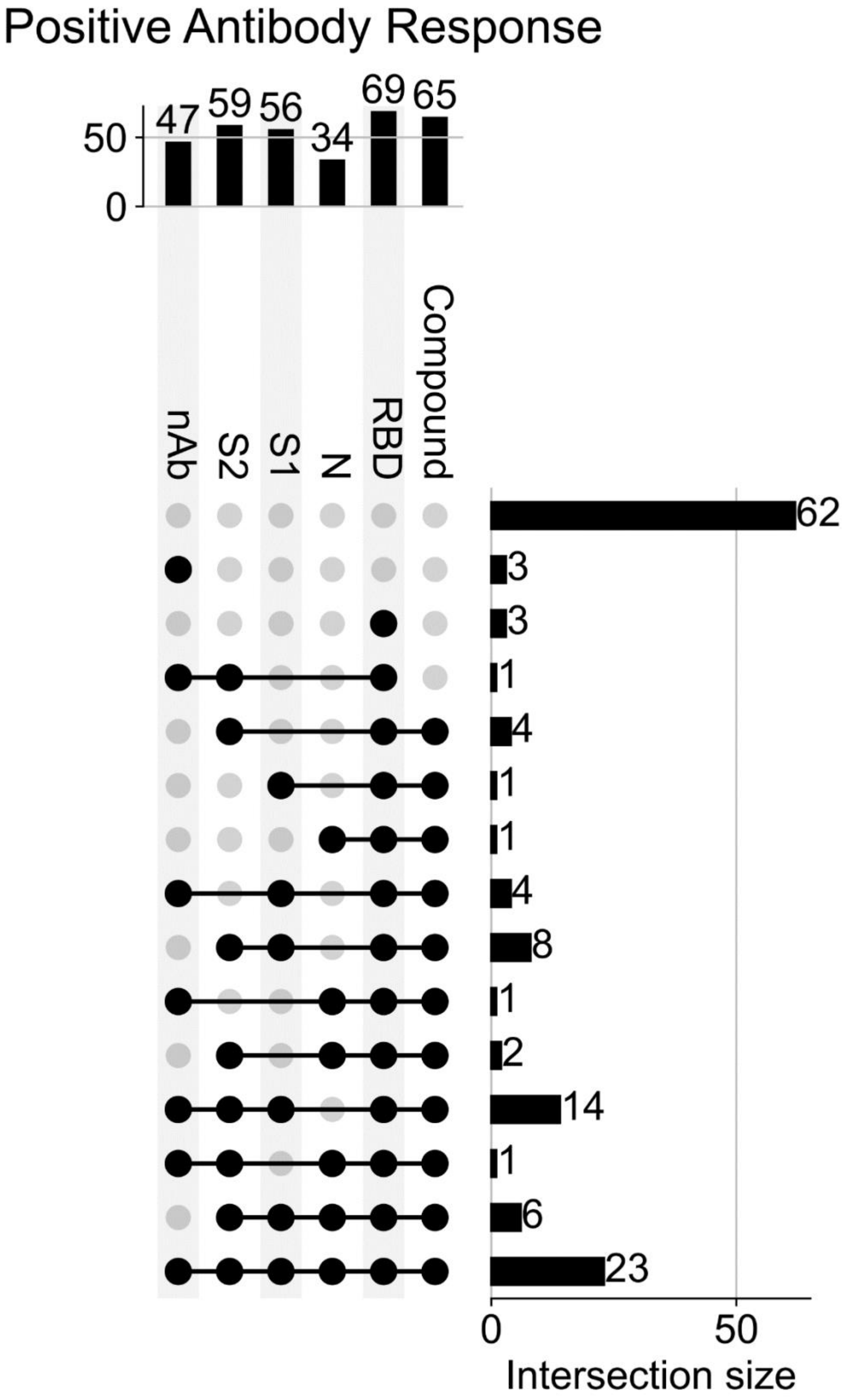

Supplementary Figure 4

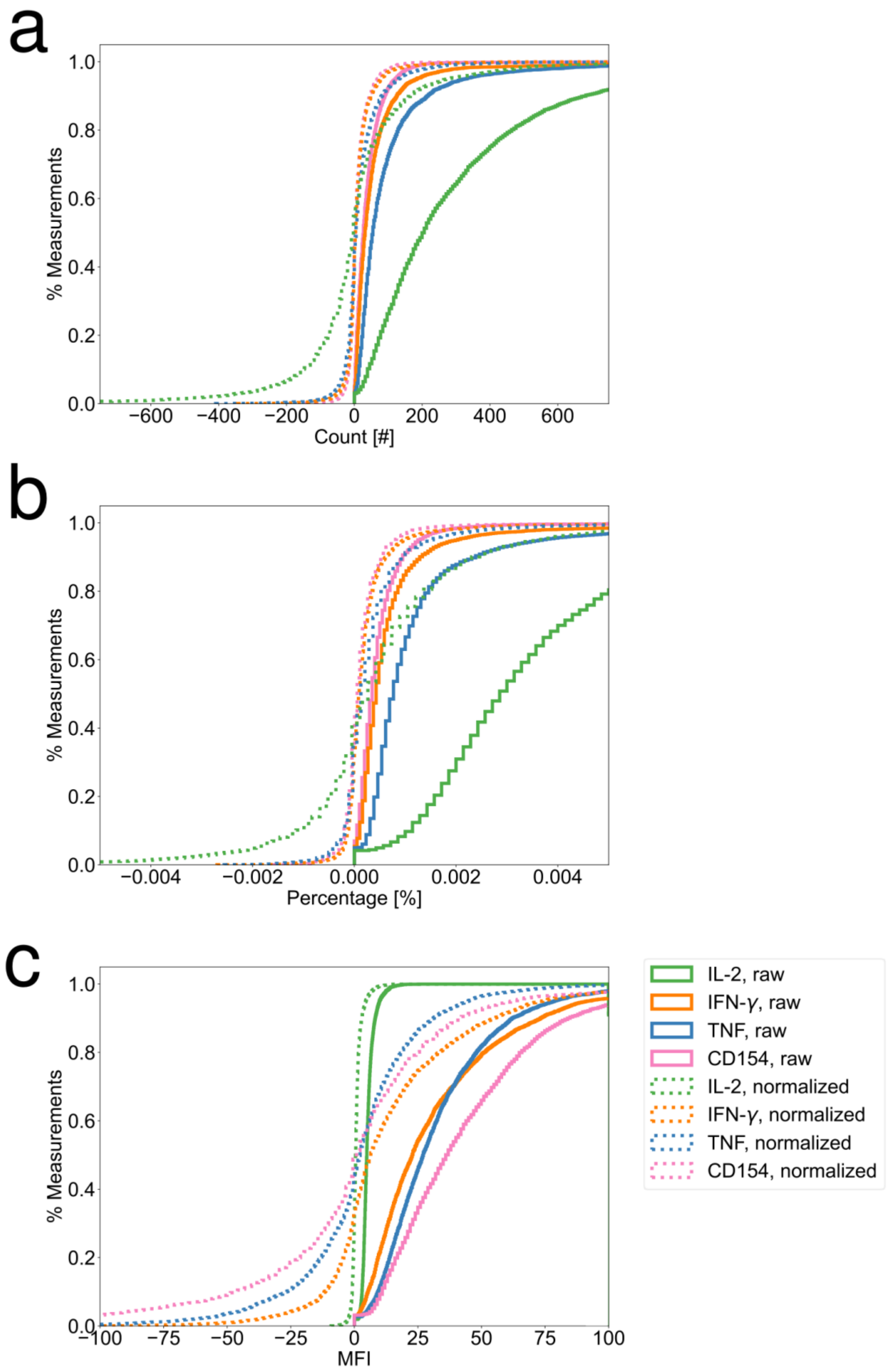

Supplementary Figure 5

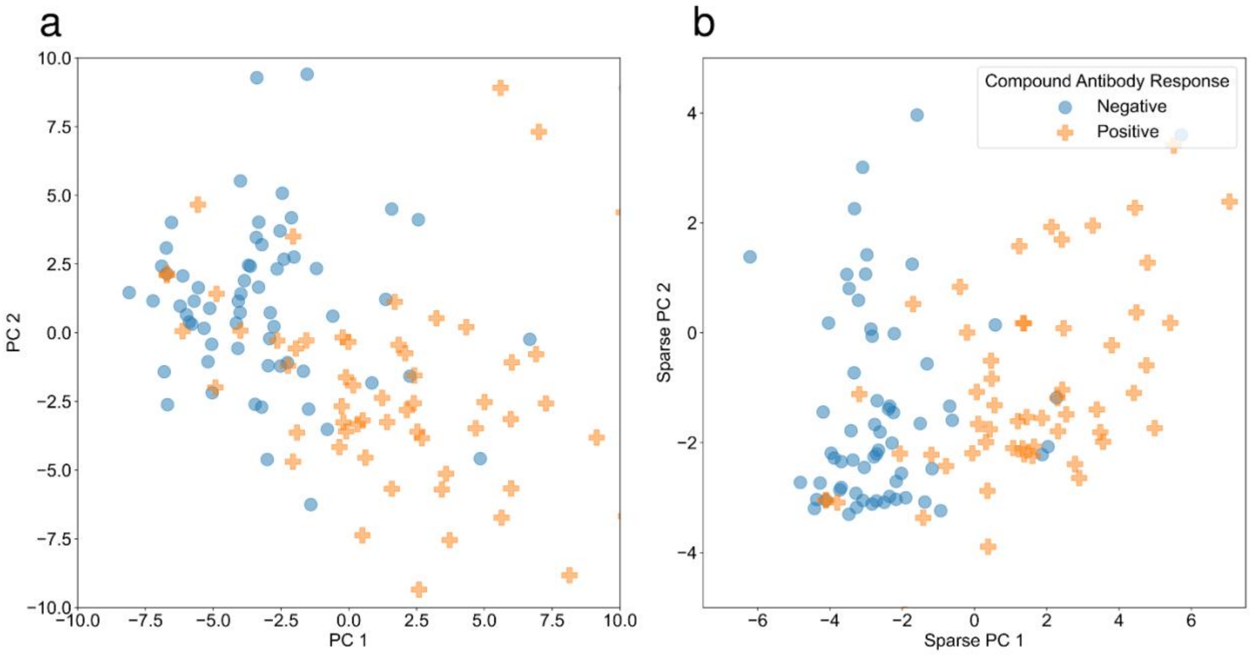

Supplementary Figure 6

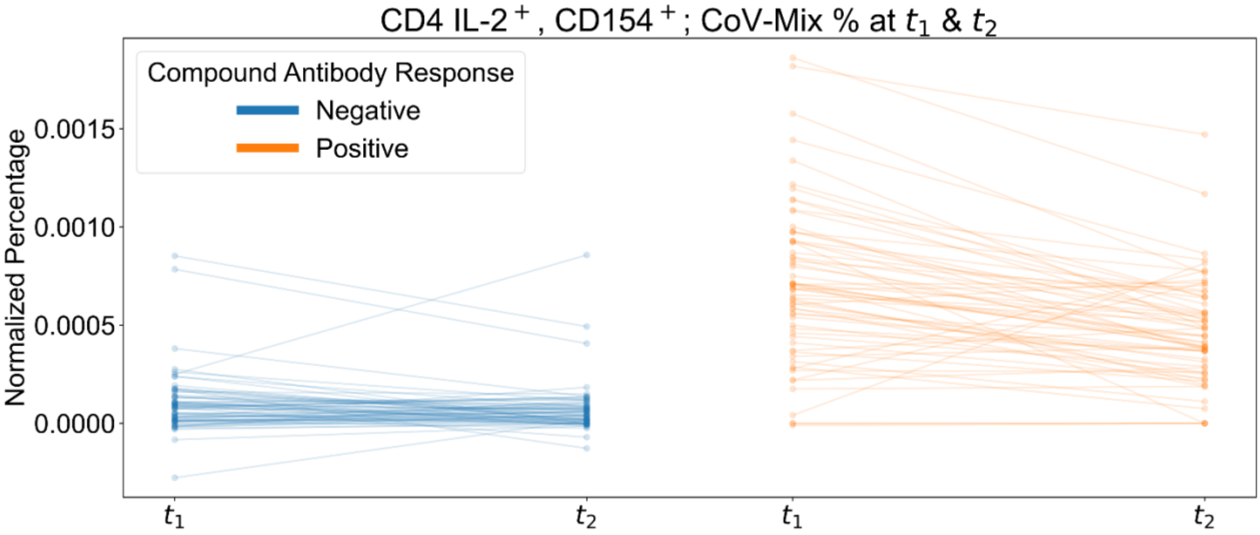

Supplementary Table 1

	SARS-CoV-2 Spike S1 IgG	SARS-CoV-2 Spike S2 IgG	SARS-CoV-2 N IgG
**Antigen**	SARS-CoV-2 (2019-nCoV) Spike S1-His Recombinant Protein (HPLC-verified)	SARS-CoV-2 (2019-nCoV) Spike S2 ECD-His Recombinant Protein	SARS-CoV-2 (2019-nCoV) Nucleocapsid-His recombinant Protein
**Coating concentration**	20 ng/well	40 ng/well	
**Blocking**	1 % BSA, overnight		0.1 % BSA, overnight
**Washing Solution**	0.05 % Tween 80		
**Sample dilutions (in assay buffer)**	1:20, 1:40; 1:100, 1:200, 1:500, 1:2’500	1:20, 1:100; 1:500, 1:2500, 1:12’500, 1:62’500	
**Plasma incubation**	1 h, at 37 °C		
**Anti-human IgG Biotin/HRP**	1:1’000	1:60’000	
**Streptavidin-poly-HRP**	1:20’000	-	
**Substrate**	3,3′,5,5′-Tetramethylbenzidin (TMB)		
**Substrate incubation**	30 min, at room temperature		
**Stop solution**	2 M H_2SO_4		

Supplementary Table 2

**Antibody Type**	**# Subjects; [% neg., % pos.]**	**Optimal Cutoff**	**Balanced Accuracy [%]**	**Sensitivity [%]**	**Specificity [%]**
RBD IgG	112; [50, 50]	[27, 67]	[96, 97]	[93, 96]	[96, 100]
N IgG	112; [50, 50]	[0.03, 6.76]	[87, 90]	[73, 91]	[82, 100]
S1 IgG	112; [50, 50]	[7, 33]	[95, 96]	[89, 93]	[96, 100]
S2 IgG	112; [50, 50]	[0.6, 8.9]	[97, 98]	[95, 96]	[98, 100]
nAb	94; [43, 57]	[5, 20]	[94, 97]	[89, 94]	[100, 100]

Supplementary Table 3

**Model**	**AUROC**	**AUPRC**
Rand. guess	0.50	0.57
LR # [-]	0.92; [0.73, 1.00]	0.93; [0.74, 1.00]
LR # [/]	0.86; [0.62, 1.00]	0.85; [0.54, 1.00]
LR % [-]	0.91; [0.69, 1.00]	0.92; [0.71, 1.00]
LR % [/]	0.91; [0.67, 1.00]	0.91; [0.67, 1.00]
LR MFI [-]	0.87; [0.64, 1.00]	0.88; [0.61, 1.00]
LR MFI [/]	0.78; [0.47, 0.99]	0.79; [0.46, 0.99]
LR #,% [-]	0.95; [0.79, 1.00]	0.95; [0.77, 1.00]
LR #,% [/]	0.93; [0.73, 1.00]	0.93; [0.72, 1.00]
GB # [-]	0.96; [0.80, 1.00]	0.96; [0.81, 1.00]
GB # [/]	0.92; [0.73, 1.00]	0.93; [0.69, 1.00]
GB % [-]	0.95; [0.76, 1.00]	0.95; [0.75, 1.00]
GB % [/]	0.92; [0.73, 1.00]	0.92; [0.67, 1.00]
GB MFI [-]	0.93; [0.73, 1.00]	0.93; [0.70, 1.00]
GB MFI [/]	0.92; [0.72, 1.00]	0.92; [0.68, 1.00]
GB #,% [-]	0.96; [0.80, 1.00]	0.96; [0.76, 1.00]
GB #,% [/]	0.93; [0.73, 1.00]	0.93; [0.72, 1.00]

Supplementary Table 4

**Assay**	**Control Treatment**	**CV, all subjects, [%]**	**CV, neg. subjects, [%]**
TNF	neg.	33	35
	pos.	41	44
IFN-γ	neg.	37	38
	pos.	40	43
IL-2	neg.	36	39
	pos.	42	44
CD154	neg.	48	50
	pos.	40	42

Supplementary Table 5

Assay	Control Treatment	CV [%]
TNF	1	38
	2	20
	7	24
IFN-γ	1	49
	2	22
	7	24
IL-2	1	55
	2	31
	7	43
CD154	1	85
	2	38
	7	33

Supplementary Table 6

**Model**	**Balanced Accuracy**	**Sensitivity**	**Specificity**
Random guess	0.50	0.49	0.51
LR #	0.83; [0.62, 1.00]	0.82; [0.45, 1.00]	0.85; [0.50, 1.00]
LR %	0.84; [0.60, 1.00]	0.86; [0.55, 1.00]	0.82; [0.44, 1.00]
LR MFI	0.79; [0.56, 0.97]	0.77; [0.40, 1.00]	0.81; [0.44, 1.00]
LR #, %	0.87; [0.69, 1.00]	0.84; [0.50; 1.00]	0.91; [0.67, 1.00]
GB #	0.90; [0.72, 1.00]	0.89; [0.56, 1.00]	0.92; [0.67, 1.00]
GB %	0.90; [0.70, 1.00]	0.89; [0.57; 1.00]	0.91; [0.62, 1.00]
GB MFI	0.86; [0.64, 1.00]	0.85; [0.53, 1.00]	0.86; [0.50, 1.00]
GB #, %	0.90; [0.70, 1.00]	0.88; [0.55, 1.00]	0.92; [0.67, 1.00]
